# Challenges of Dementia Care among Older Adults Living Alone in Japan: Insights from a Memory Clinic Study

**DOI:** 10.31662/jmaj.2025-0422

**Published:** 2025-12-26

**Authors:** Shinichiro Maeshima, Aiko Osawa, Chiaki Kamizato, Hidenori Arai

**Affiliations:** 1 Education and Innovation Center, National Center for Geriatrics and Gerontology, Obu, Japan

**Keywords:** older adults, living alone, caregiver burden, long-term care insurance, dementia, cognitive function

## Abstract

**Introduction:**

Japanese society is rapidly aging, and increasing numbers of older adults are living alone, which may hinder the early detection and management of dementia. This study aimed to investigate the characteristics of older adults living alone who attended a memory clinic, focusing on cognitive, physical, and caregiving burdens.

**Methods:**

A total of 278 older adults (aged 66-94 years) were categorized as living alone (n = 57) or living with others (n = 221). Evaluations included cognitive, physical, and behavioral assessments, caregiver burden (Zarit Burden Interview-8), and long-term care insurance (LTCI) status.

**Results:**

The group living alone was older and included a higher proportion of women. They had lower cognitive scores on the Montreal Cognitive Assessment-Japanese version and the Raven’s Colored Progressive Matrices but showed higher grip strength, Rivermead Mobility Index, and Vitality Index scores. Walking speed, Timed Up and Go Test, and depressive symptoms were similar between groups. Caregivers of those living with others reported greater role strain, while LTCI use was generally low, particularly among participants living alone.

**Conclusions:**

Older adults living alone face a greater risk of cognitive decline despite preserved physical function. Low LTCI utilization highlights the need for enhanced support. Effective strategies should aim to maintain both cognitive and physical function while addressing caregiver needs across different living arrangements.

## Introduction

As of August 1, 2024, Japan’s total population was 123.88 million, of which 36.25 million individuals were aged 65 years or older, accounting for 29.3% of the total population ^(1)^. Japan is among the world’s longest-living nations, with an average life expectancy of 81.1 years for men and 87.1 years for women in 2023, and these figures are projected to continue rising ^(2)^. Concurrently, the number of older adults living alone is also increasing. In 2020, 15.0% of men and 22.1% of women aged 65 years and older lived alone, and these proportions are projected to rise to 26.1% and 29.3%, respectively, by 2050. Such demographic changes are expected to have profound implications for dementia care and support ^(3)^.

The prevalence of dementia increases sharply with age, and the number of individuals with dementia in Japan is estimated to reach 4.5 million by 2025. Living alone has been associated with an increased risk of cognitive decline, delayed diagnosis, and inadequate post-diagnostic support, as well as heightened risks of loneliness, depression, and social isolation ^(4), (5)^. From a caregiving perspective, older adults living alone often rely on their adult children rather than their spouses, which may create unique challenges due to geographical distance and competing responsibilities ^(6)^. However, despite the growing number of older adults living alone, few studies have systematically examined their clinical characteristics and caregiving burden in the context of specialized memory clinics in Japan ^(7)^. Moreover, relatively few studies have explored the role of social support systems, such as the long-term care insurance (LTCI) program, which plays a central role in dementia care in Japan.

The present study aimed to characterize the clinical and functional profiles of older adults living alone who attended a memory clinic and to assess the caregiving burden experienced by their primary caregivers. Additionally, from a social support perspective, we examined the participants’ LTCI application and certification status. By comparing those living alone with those living with others, we sought to identify the specific risks and needs of this vulnerable population and to inform tailored, effective dementia care strategies.

## Materials and Methods

This study included 278 patients (124 men and 154 women) who visited a memory clinic between June 2023 and December 2024. Participants were aged 66-94 years (mean age: 79.9 ± 5.9 years) and had education levels ranging from 6 to 21 years (mean ± standard deviation: 11.5 ± 2.5 years). Medical diagnoses were made according to the Diagnostic and Statistical Manual of Mental Disorders, Fifth Edition, published by the American Psychiatric Association, using the diagnostic criteria for major neurocognitive disorders (dementia) and minor neurocognitive disorders (mild cognitive impairment [MCI]) ^(8)^. The results demonstrated that 161 patients had dementia, 89 had MCI, and 28 had normal cognitive function. The distribution of dementia subtypes was as follows: Alzheimer’s disease, 133 cases (including 18 with cerebrovascular disease); vascular dementia, 16 cases; Lewy body dementia, five cases; and frontotemporal dementia; seven cases.

Participants were classified into two groups: those living alone (independently, without cohabitants) and those living with others (cohabiting with family). Intergroup comparisons were conducted for demographic characteristics, cognitive and physical functions, behavioral symptoms, caregiver burden (assessed using the Zarit Burden Interview-8 [ZBI-8]), and LTCI utilization.

1. Background factors: age, sex, and years of education

2. Physical function assessment:

(a) Muscle strength: grip strength of the dominant hand

(b) Mobility: Timed Up and Go Test ^(9)^, walking speed, and Rivermead Mobility Index ^(10)^

(c) Activities of daily living: Barthel Index ^(11)^

3. Cognitive function assessment:

(a) Montreal Cognitive Assessment ^(12)^

(b) Raven Colored Progressive Matrices ^(13)^

(c) Frontal Assessment Battery ^(14)^

4. Behavioral and psychological symptoms:

(a) Dementia Behavioral Disturbance Scale ^(15)^

(b) Geriatric Depression Scale ^(16)^

(c) Vitality Index ^(17)^

5. Caregiver burden and service utilization assessment:

(a) ZBI-8 ^(18)^ administered to family caregivers

(b) Utilization of the LTCI system: Application status and certification level (support or care) were confirmed through interviews with patients and/or their primary supporters.

### Statistical analysis

Statistical analysis was performed using JMP 17.0 (SAS Institute, Cary, NC, USA). For comparisons between the groups living alone and living with others, the Mann-Whitney U test was applied to continuous variables, and the chi-square (χ^2^) test was applied to categorical variables, with a significance level set at p < 0.05.

This study was conducted in accordance with the Declaration of Helsinki and was approved by the Institutional Review Board of the National Center for Geriatrics and Gerontology (Approval No. 1658-2). Owing to its retrospective design, written informed consent was not obtained from individual participants. Instead, an opt-out procedure was implemented, and study information was disclosed on the institutional website, allowing patients and their families the opportunity to decline participation.

## Results

The living-alone group included 57 participants, while the living with others group comprised 221 participants (Table 1). The living-alone group was older and included a higher proportion of women. No significant differences were observed in the distribution of dementia, MCI, or cognitively robust individuals between the two groups.

**Table 1.** Comparison of Participants Living Alone Versus Those Living with Others.

	Living alone	Living together	p
	N=57	N=221	
Age, years old	83 (72-92)	80 (66-94)	0.0022
Gender, men/women	13/44	111/110	0.0001†
Education years, years	11 (6-20)	12 (6-21)	0.0435
Etiology (Dementia/MCI/Robust)	1940/12/5	121/77/23	0.2083†

Grasping power right, kg	18 (8.9-36)	21 (7-42.6)	0.0139
Grasping power left, kg	14 (2-34)	19.3 (6-45.7)	<.0001
Walking speed, m/sec	0.97 (0.4-1.38)	0.94 (0.24-1.47)	0.2312
Timed Up and Go Test, sec	12.8 (8.5-22.8)	11.5 (6.8-30.3)	0.076
Rivermead Mobility Index, /15	13 (3-15)	14 (5-15)	0.0014
Barthel index, /100	100 (20-100)	100 (15-100)	0.4239

Montreal Cognitive Assessment, /30	14 (4-27)	17 (1-30)	0.0022
Raven’s Colored Progressive Matrices, /36	21.5 (0-36)	25 (0-36)	0.005
Frontal Assessment Battery, /18	9 (1-14)	10 (2-17)	0.1549
Dementia Behavior Disturbance Scale, /112	11 (1-33)	12 (0-76)	0.3321
Geriatric Depression Scale, /15	3 (0-14)	3 (0-14)	0.5823
Vitality index, /10	10 (6-10)	9 (2-10)	0.0516

Number of people living together	1 (1-1)	2 (2-8)	<.0001
Key Person	Son/Daughter 47 Other 7	Spouse 134 Son/Daughter 78 Other 7	<.0001

Median (range), Mann-Whitney U test, †Chi-square test

Regarding physical function, walking speed and Timed Up and Go Test scores did not differ between the groups, whereas grip strength and Rivermead Mobility Index scores were significantly higher in the living-alone group. In contrast, cognitive function scores were significantly lower in the living-alone group for both Montreal Cognitive Assessment-Japanese version and Raven Colored Progressive Matrices. No significant differences were identified in the Dementia Behavioral Disturbance Scale, Geriatric Depression Scale, or Vitality Index scores.

**Figure 1.**
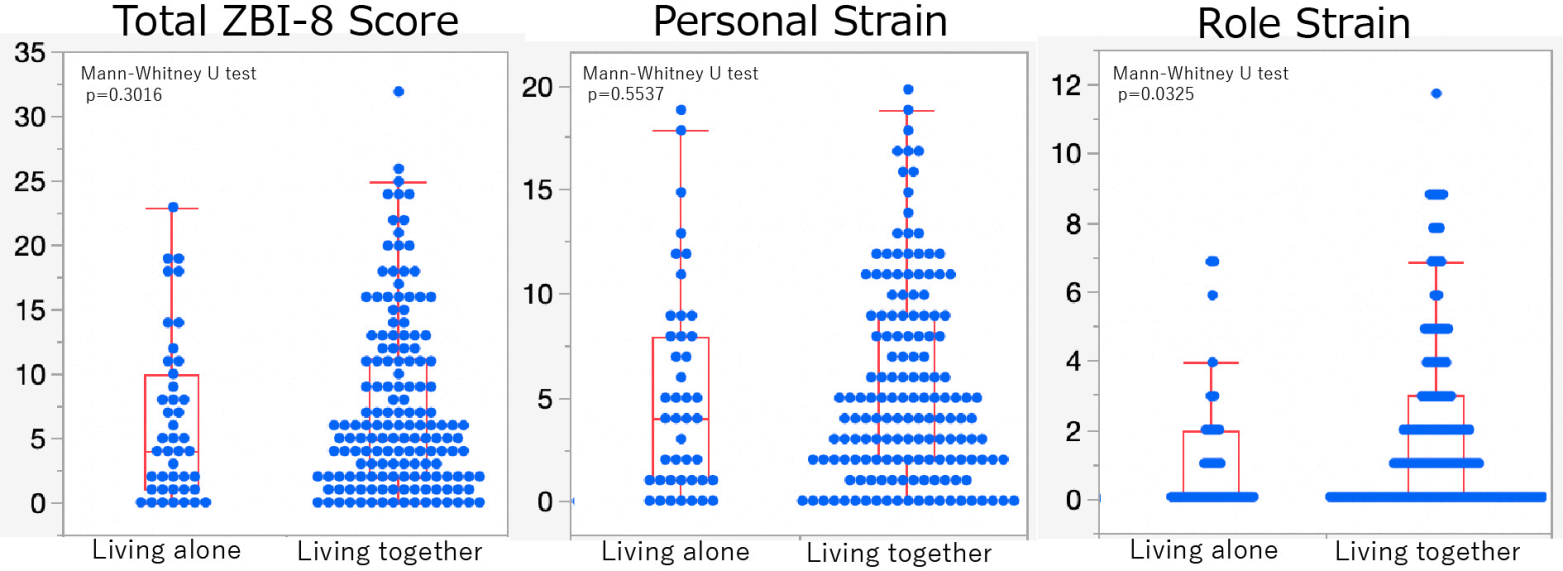
Comparison of caregiver burden (assessed using ZBI-8) between caregivers of older adults living alone and those living with others. No significant differences were observed in the total ZBI-8 score or personal strain subscale scores between the two groups. However, the role-strain subscale score was significantly higher among caregivers of older adults living with others. ZBI-8: Zarit Burden Interview-8.

Caregiver burden, assessed using the ZBI-8, did not differ significantly between groups in terms of total scores or personal strain. However, role strain was greater among caregivers of participants living with others (Figure 1).

**Figure 2.**
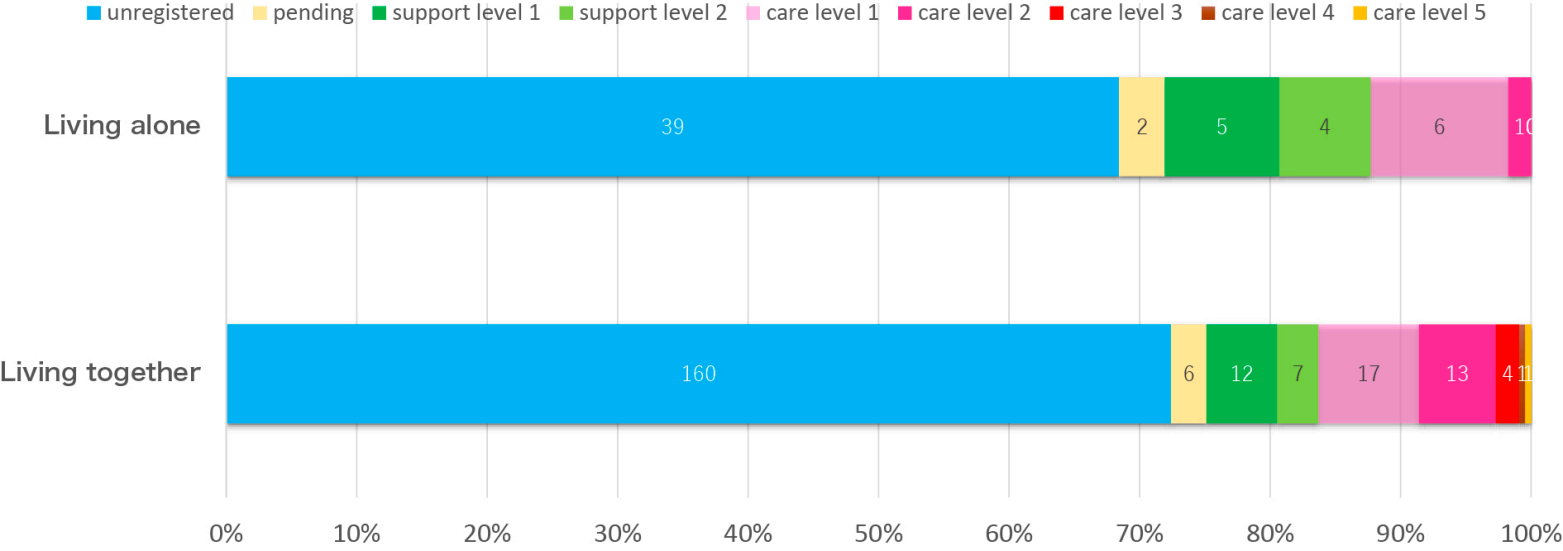
Distribution of LTCI certification status by living arrangement. Distribution of LTCI applications and certification status among older adults living alone (n = 57) and those living with their families (n = 221). Categories include unregistered, pending certification, support levels 1-2, and care levels 1-5. A substantial proportion of participants, particularly in the living-alone group, did not apply for LTCI at the time of assessment. LTCI: long-term care insurance.

With respect to LTCI, 78 participants (28%) applied for certification: 28 were classified as requiring support, 42 as requiring nursing care, and eight were awaiting assessment. Among those living with others, 160 were unregistered, six were pending, and 55 were certified; among those living alone, 39 were unregistered, two were pending, and 16 were certified. The detailed distribution of support and care levels is illustrated in Figure 2.

## Discussion

This study revealed that older adults living alone exhibited significantly lower cognitive function than those living with others, despite no differences in diagnostic distribution. These results are consistent with previous reports suggesting that solitary living reduces opportunities for cognitive stimulation and social interaction, thereby accelerating the decline in executive and memory functions ^(5), (19), (20)^. Social and environmental factors, rather than disease status alone, may thus contribute to these differences.

Conversely, physical function and depressive symptoms were comparable across the groups, with the living-alone group demonstrating higher vitality and mobility scores. These findings may reflect adaptive strategies, such as maintaining independence through daily household activities that provide both physical and motivational stimulation. Previous studies have indicated that autonomy in daily life can preserve physical function and foster resilience, even in the context of cognitive impairment ^(21), (22)^. Thus, living alone may entail both an increased risk of cognitive decline and potential benefits in sustaining physical independence.

Regarding caregiver burden, primary caregivers of individuals living alone reported lower role strain compared with those caring for cohabiting individuals. This difference is likely attributable to the fact that cohabiting caregivers, often spouses, face constant care demands, whereas the caregivers of individuals living alone are frequently adult children providing intermittent or long-distance care. Personal strain, however, was comparable between the groups, indicating that the emotional burden of caregiving remained substantial regardless of living arrangements. These findings emphasize the importance of tailoring support to both patients and caregivers, considering the household structure and caregiver relationships ^(6), (23)^.

From clinical and policy perspectives, these findings highlight the need for memory clinics and community services to enhance early detection strategies for older adults living alone. Interventions should aim to balance the risks of cognitive decline with the adaptive strengths associated with independent living. Community-based programs that encourage social participation, preserve daily autonomy, and support distant caregivers may also prove effective.

This study had several limitations that must be acknowledged. First, the study’s cross-sectional design precludes causal inferences. Second, the study did not assess the proximity or accessibility of family members, which may have influenced caregiving burden and patient independence. Third, the LTCI utilization was low, limiting the evaluation of its potential protective effects. Fourth, depressive symptoms were assessed only through self-reporting, which may have underestimated psychological distress. Fifth, our cohort included a small number of dementia subtypes other than Alzheimer’s disease, such as dementia with Lewy bodies and frontotemporal dementia, which may have introduced diagnostic heterogeneity. However, this also reflects the actual spectrum of patients encountered in memory clinics, making our findings clinically relevant to real-world practice. Finally, this single-center study has limited generalizability. Future research should employ longitudinal, multiregional designs and incorporate broader psychosocial measures, including loneliness, social networks, caregiver relationships, and formal service use. Furthermore, longitudinal follow-up data were excluded; accumulating such data will be essential to elucidate the trajectories of cognitive and functional changes among older adults living alone. We plan to obtain follow-up information in future studies.

### Conclusion

This study demonstrated that older adults in the living-alone group exhibited reduced cognitive function but maintained physical vitality and abilities, indicating both risk and resilience. Caregivers in the living with others group experienced greater role strain; however, emotional burden was universal. These findings underscore the need for community-based, individualized care strategies tailored to the needs of both individuals living alone and those living with others.

## Article Information

### Acknowledgments

We thank all participants for their valuable contributions to this study.

### Author Contributions

Study conception and design: Shinichiro Maeshima. Data collection: Shinichiro Maeshima and Chiaki Kamizato. Data analysis: Shinichiro Maeshima. Supervision and data interpretation: Hidenori Arai. Discussion and critical review of the manuscript: Aiko Osawa and Hidenori Arai. Manuscript drafting: Shinichiro Maeshima. All authors have read and approved the final manuscript and agree to be accountable for all aspects of the work.

### Conflicts of Interest

None

### Approval Code Issued by the Institutional Review Board (IRB) and the Name of the Institution That Granted the Approval

This study was approved by the Ethics Committee of the National Center for Geriatrics and Gerontology (1658-2).
